# Combined assembly of long and short sequencing reads improve the efficiency of exploring the soil metagenome

**DOI:** 10.1186/s12864-021-08260-3

**Published:** 2022-01-07

**Authors:** Guoshun Xu, Liwen Zhang, Xiaoqing Liu, Feifei Guan, Yuquan Xu, Haitao Yue, Jin-Qun Huang, Jieyin Chen, Ningfeng Wu, Jian Tian

**Affiliations:** 1 Biotechnology Research Institute, Chinese Academy of Agricultural Sciences, No.12 Zhongguancun South Street, Beijing, 100081 People’s Republic of China; 2 Department of Biology and Biotechnology, Xinjiang University, 666 Shengli Road, Urumqi, 830046 People’s Republic of China; 3 State Key Laboratory for Biology of Plant Diseases and Insect Pests, Institute of Plant Protection, Chinese Academy of Agricultural Sciences, Beijing, 100193 People’s Republic of China

**Keywords:** Soil DNA, Metagenome, PacBio, Illumina, Combined assembly

## Abstract

**Background:**

Advances in DNA sequencing technologies have transformed our capacity to perform life science research, decipher the dynamics of complex soil microbial communities and exploit them for plant disease management. However, soil is a complex conglomerate, which makes functional metagenomics studies very challenging.

**Results:**

Metagenomes were assembled by long-read (PacBio, PB), short-read (Illumina, IL), and mixture of PB and IL (PI) sequencing of soil DNA samples were compared. Ortholog analyses and functional annotation revealed that the PI approach significantly increased the contig length of the metagenomic sequences compared to IL and enlarged the gene pool compared to PB. The PI approach also offered comparable or higher species abundance than either PB or IL alone, and showed significant advantages for studying natural product biosynthetic genes in the soil microbiomes.

**Conclusion:**

Our results provide an effective strategy for combining long and short-read DNA sequencing data to explore and distill the maximum information out of soil metagenomics.

**Supplementary Information:**

The online version contains supplementary material available at 10.1186/s12864-021-08260-3.

## Background

Metagenomics studies have revealed that in soil microbiomes, uncultured species outnumber the culturable by two to three orders of magnitude [1], highlighting the vast potential of high-throughput DNA sequencing to discover novel functional genes and pathways directly from the soil samples. To avoid duplication of previous research and to discover genes/enzymes with novel applicable bioactivities and/or physiochemical properties [2, 3], it is important to direct attention to environments and microbes that remain unexplored.

High-throughput, short-read DNA sequencing platforms such as the Illumina are usually referred to as “second-generation” sequencing technologies, are currently employed in metagenomics [4–7]. The development of long-read “third-generation” sequencing technologies such as those developed by Pacific Biosciences (PacBio) (PB) and Oxford Nanopore, coinciding with the more advanced bioinformatics tools, provide rapid, affordable DNA sequencing and assembly of long reads from microbial consortia [6, 8–10]. A number of studies have applied either the second- or third- generation sequencing technologies in metagenomic studies. Hybrid approaches to metagenomic assembly have yielded better results [11, 12]. Systematic evaluation of the performance of these technologies on the quality of metagenomic sequences is urgently needed.

In the microbial metagenome, an important class of genetic resources include the genes that encode bioactive natural products (NPs) [5, 13]. These small molecules possess a wide range of biological activities [5], and comprise the greatest source of unexplored chemotherapeutics such as antimicrobials, anticancer agents, and immunomodulators for pharmaceutical, agricultural, and food processing applications [14, 15]. Surveys of microbial biosynthetic diversity across environmental samples have revealed enormous reservoirs of untapped natural products diversity [5, 8, 16–21]. In the face of an increasing need for new therapeutics, the advent of techniques that permit the mining and expression of biosynthetic gene “cassettes “directly from microbiomes may well be the new frontier for natural product discovery [5, 13, 15, 22–24]. However, the high degree of sequence similarity and the repetition of biosynthetic domains can complicate the assembly of relatively long biosynthetic gene clusters (BGC) from metagenomic data. This has limited the straightforward application of metagenomics in natural products discovery [25].

**Fig. 1:**
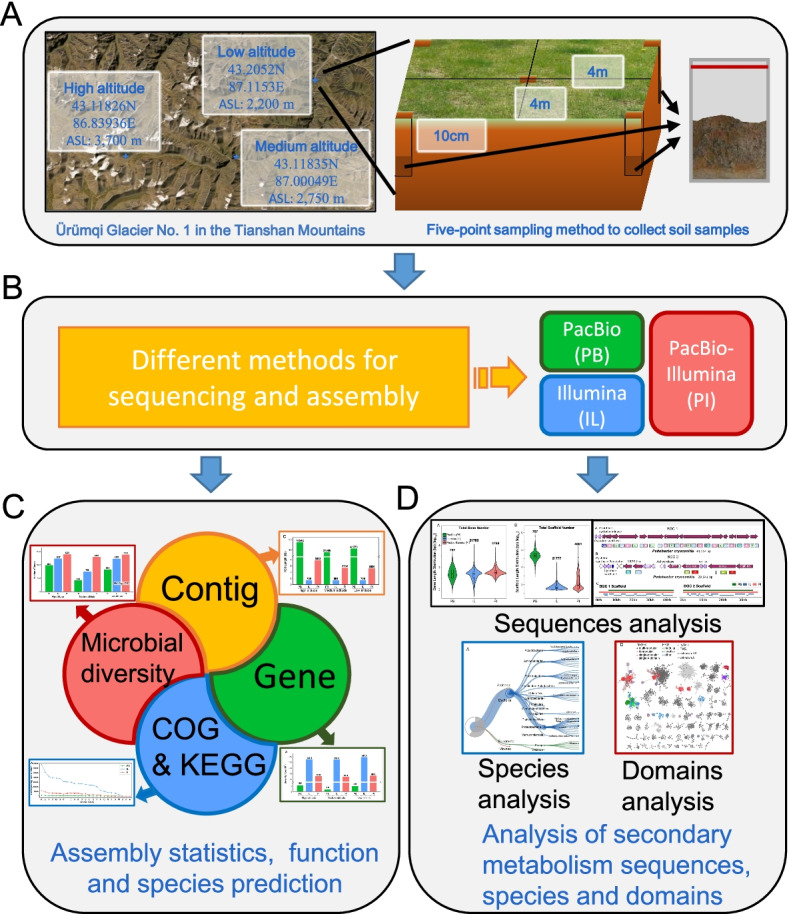
Schematic diagram of sample collection and analysis. The location and sampling method of soil samples (**A**). The samples were sequenced according to different sequencing platforms to generate three sequencing data sets (PB, IL, PI) (**B**), compared the assemble performance (**C**), and performed the secondary metabolism analysis (**D**)

As shown in Fig. 1, we applied second- and third-generation sequencing platforms (Illumina Hiseq 2000 and PacBio RS II) to sequence the soil samples that collected at low (2200 m), medium (2750 m), and high (3700 m) altitude locations in the Tianshan Mountains in Xinjiang, China. The metagenomes were assembled from the sequenced data from PacBio (PB), Illumina (IL), and the combined data from PB and IL (PI), respectively. The main objectives of the current study were to: 1) evaluate the quality of metagenomes assembled by the PB, IL, and PI approaches; 2) compare the advantages of functional metagenomics studies among the PB, IL, and PI assemblies; 3) employ the case of developing BGCs, to prove the optimal assembly strategy for the functional metagenomics research on the microbial communities of soil environments.

## Results

### PacBio, Illumina, and combined sequencing assembly statistics

**Fig. 2:**
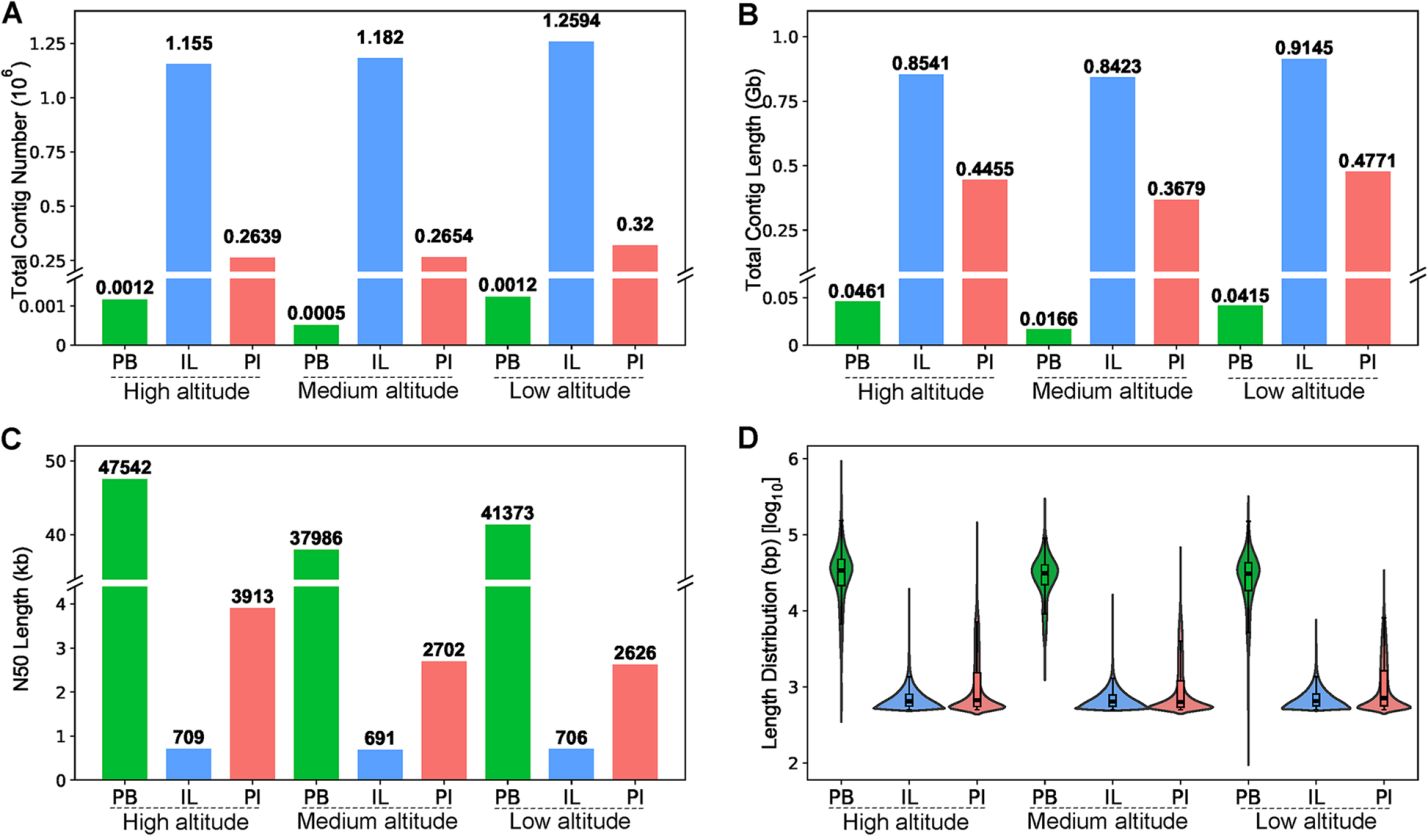
Parameters of the third-generation sequencing assemblies (SMRT, Pacific Bioscience), second-generation sequencing assemblies (Hiseq2000, Illumina), and the combined assembly for the three soil samples, including total contig number (**A**), total contig length (**B**), N50 length (**C**), and contig length distribution (**D**)

DNA extracted from three soil samples collected from the Ürümqi Glacier No. 1 in the Tianshan Mountains at 2200, 2750 and 3700 m altitudes was sequenced using both PB and IL platforms. The general information for each sample, including metagenome size (Clean Data Length), number of contigs or scaffolds, GC content, and scaffold N90 values are listed in Table S1. The IL platform out-performed PB with respect to sensitivity, which was exemplified by the total number and length of the contigs (Fig. 2A-B). However, the PB read lengths were much longer, with N50 lengths ranging from 37,986 to 47,542 bp, and the longest single read was 607,831 bp. The PB reads were mostly 5000 to 100,000 bp long (92.63%). In contrast, the IL contigs from the three soil samples had N50 lengths of 709, 691, and 706 bp. The majority of the contigs (89.53%) were shorter than 1000 bp (Fig. 2C-D, Table S1).

The longer PB reads facilitated the profiling of longer genes. Thus, the libraries from the two platforms were combined and assembled, which achieved better sensitivity and integrity of the soil DNA (Fig. 2). Combining the two sequencing libraries increased the length distribution of the contigs with N50 lengths of 3913, 2702, and 2626 bp for the soil samples collected at high, medium, and low altitudes, respectively (Fig. 2C-D). Compared with IL, the number of contigs > 1000 bp in length increased by 17.14–25.67%, and compared with PB, PI generated more contig number and longer total contig length. The GC content of the PacBio reads (61.32–65.19%) was lower than that of the Illumina contigs (64.20–65.52%) (Table S1). This is because the short reads with higher GC content might be difficult to assemble, and the contigs contained many unassembled GC-rich reads. The GC contents of the PI ranged from 62.01–64.27%, harnessing thereby the unique advantages of the two sequencing methods.

In addition, the clean data in PB (6.62–10.90 Gb) are bigger than the one in IL (4.53–4.54 Gb) (Table S1). We normalized the contig number with clean data size and found that, using per unit of clean data (1 Gb), IL could generate 264,364 ± 9774 contigs/Gb and PB only generated 108 ± 24 contigs/Gb (Fig. S1A). This result again indicated that the IL platform is more sensitive than PB in the number of contigs. The PI has larger clean data, but the contig number of PI per unit of clean data assembled (21,512 ± 1645) is still less than IL performance (Fig. S1A).

### Gene prediction from the assembled contigs

**Fig. 3:**
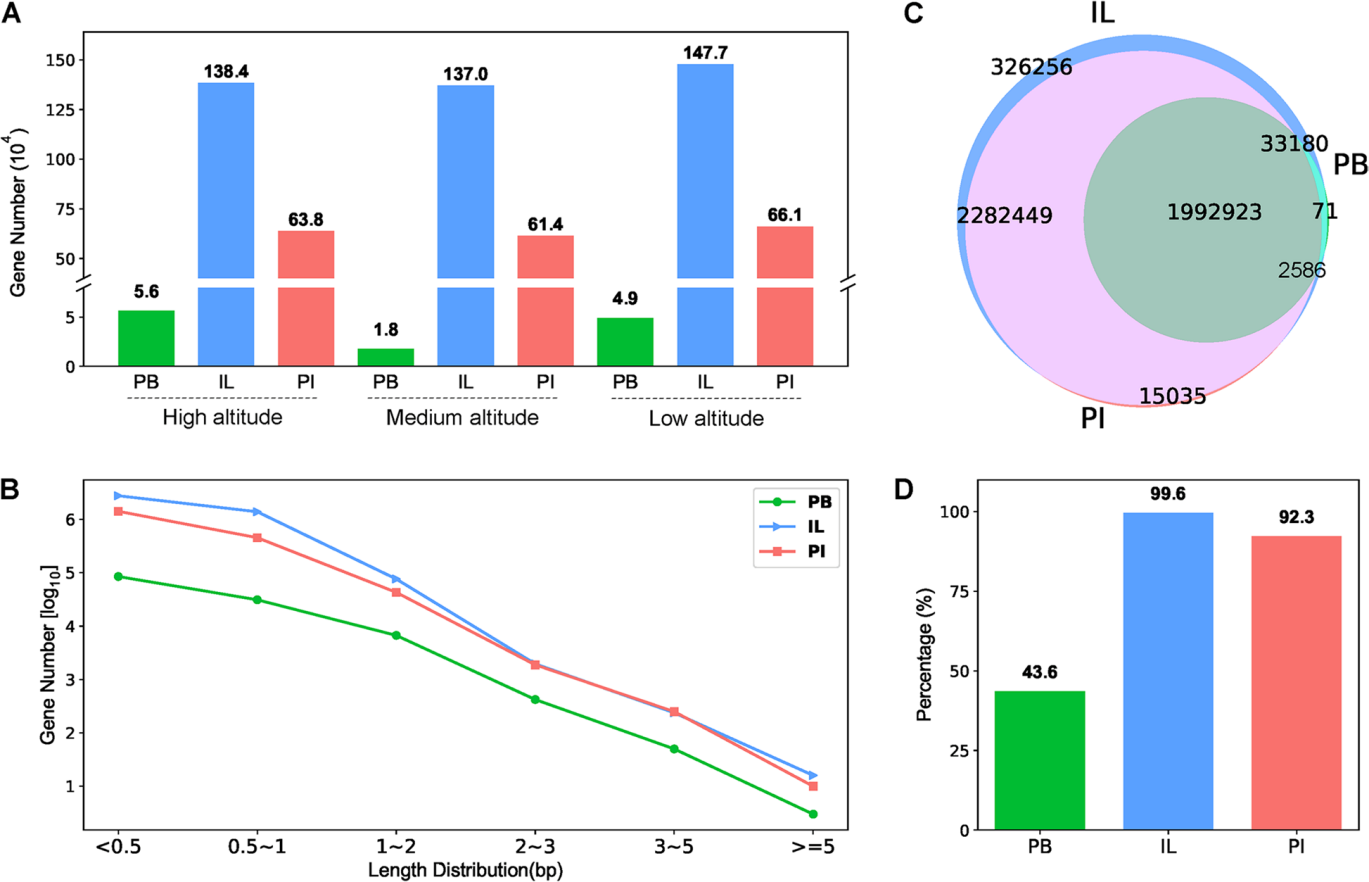
Predicted gene numbers (**A**) and gene length distributions (**B**) and the coverage of predicted genes (**C**-**D**) for the different soil samples using the three sequencing/assembly methods. The predicted protein sequences were used to perform orthologous gene analysis, and a Venn diagram was drawn to show the overlap between the three assembly methods (**C**). The percentage of total genes covered by the three sequencing methods is shown in **D**

The assembly performance of the three different methods was further evaluated by examining the predicted genes. The Illumina platform performed better in terms of sensitivity compared to PB, as shown by gene numbers (Fig. 3A) and total gene lengths (Table S2), while PB sequencing gave a larger proportion of longer gene sequences (Fig. 3B, S2, Table S3), the proportion of gene sequences number longer than 1000 bp was 5.82% in PB, while IL was 1.86%. Only third-generation sequencing assemblies for the high-altitude sample resulted in a 3.18-fold (PB, medium altitude) increase in the number of genes (Fig. 3A). This showed that the PB method had relatively unstable sequencing results. According to the gene length distribution line graph (Fig. 3B, S2, Table S3), except the high-altitude sample, the PB and PI exceeded the IL assembly by 7.34- and 2.14-fold, respectively, in the number of genes with lengths ≥2000 bp. In addition, the number of genes ≥2 kb in the PI was 2142, compared to 2214 and 474 in the IL and PB assemblies, respectively.

The orthologous genes were analyzed to compare the common and unique genes predicted by the three assembly methods. The genes from the PB assembly accounted for only 43.60% of the total gene pool, while the genes from the IL and PI assemblies accounted for 99.62 and 92.27% of the total genes, respectively (Fig. 3C-D). The PI covered 82.06, 82.53 and 82.57% of the total gene set in the high, medium, and low altitude samples, respectively. The 151,923, 157,139, and 137,589 genes in the three samples were only revealed by the PI assembly (Fig. S3). However, the Venn diagram also pointed out genes that were lost from the PI assembly predicted by the contigs, resulting from assembly errors.

### Annotation of the predicted genes

**Fig. 4:**
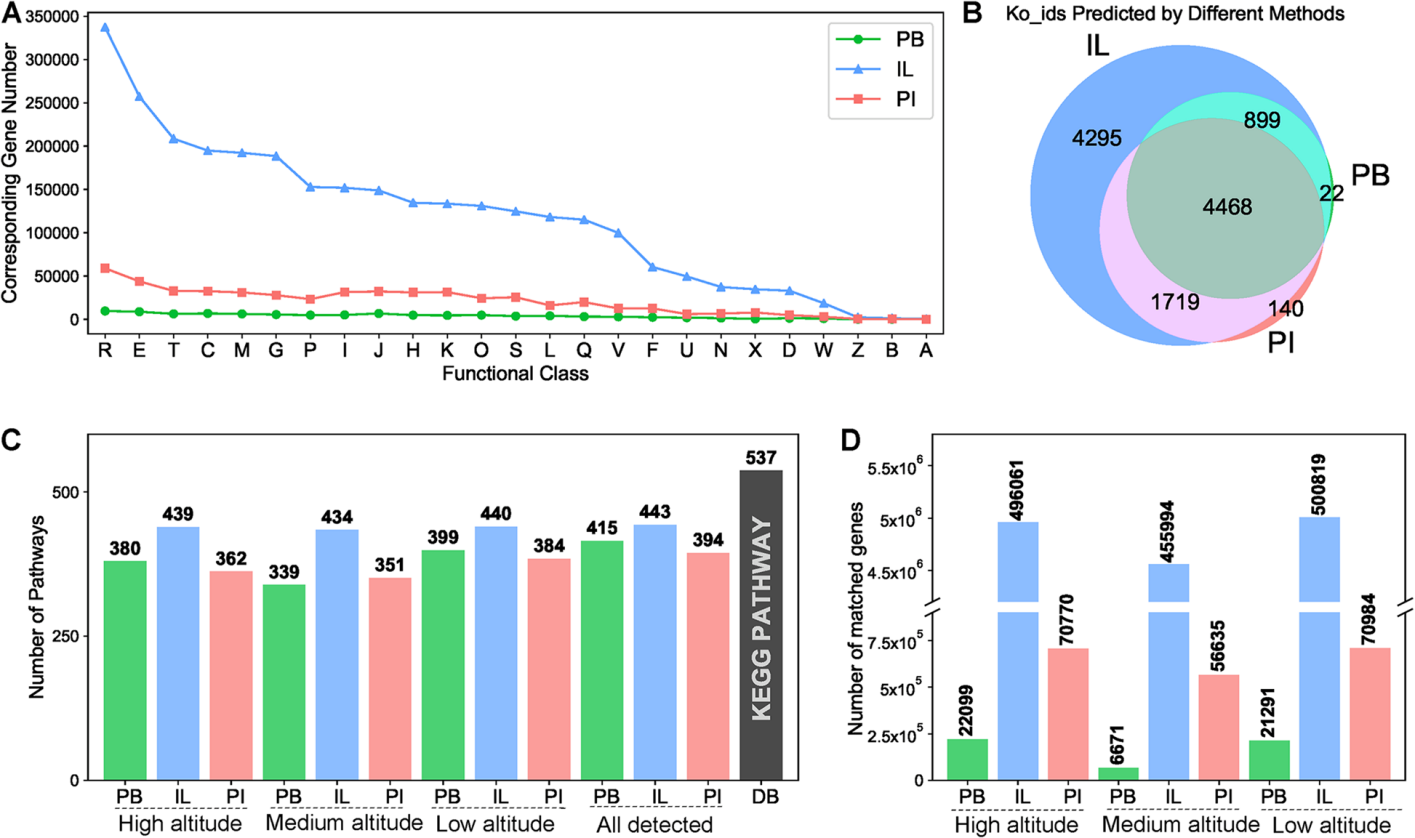
Gene functional annotation determined using the COG (cluster of orthologous group) and KEGG (Kyoto Encyclopedia of Genes and Genomes) databases. The numbers of clusters and Ko-ids obtained by each method were plotted collectively (**A** and **B**). Pathways predicted by the three sequencing/assembly methods and the number of pathways in the KEGG [26–28] pathway database (**C**), and the number of genes corresponding to pathways predicted by the three sequencing/assembly methods and their proportion in each predicted gene (**D**). In the x-axis of panel **C**, “All” indicates the average number of pathways predicted in the DNA extracted from the high, medium, and low altitude soil samples. “DB” refers to the KEGG Pathway Database (url: https://www.genome.jp/kegg/pathway.html#environmental). The detailed function classes in Fig. 4A are described in supplementary materials Table S4 and the detail metabolic pathways were predicted using ipath 3 (url: https://pathways.embl.de/ [29]) and are shown in Fig. S5. The curves in panels A are arranged in descending order of PI data

The predicted genes were annotated using the COG and KEGG [26–28] databases to evaluate the quality and quantity of the predicted proteins from the three sequencing/assembly strategies. As shown in Fig. 4A and Fig. S4, the functional gene distribution of the three assemblies were similar, i.e., they were all enriched in genes involved in energy production and conversion (C), transport and metabolism of amino acids (E), carbohydrates (G), coenzymes (H), Inorganics (P) and lipids (I), translation, ribosomal structure and biogenesis (J), transcription (K), and signal transduction (T) (Fig. 4A). However, the PI assembly showed stable functional gene numbers (PB: 31,772 ± 13,546 vs. IL: 975,330 ± 31,417 and PI: 171,836 ± 14,892), and in general, the number were higher in the IL and PI assemblies than in the PB assembly (Fig. S4).

Metabolic pathway analysis based on the KEGG database suggested that all three strategies covered most of the predicted Ko-ids (38.70%) (Fig. 4B, S5A-C). There were total of 537 pathways in the KEGG Pathways database, although PI assembly predicted 394 (366 ± 14) pathways, less than the 443 (438 ± 3) predicted pathways for IL, and 415 (373 ± 25) predicted pathways for PB assembly, PI assembly results was more stable than PB assembly (Fig. 4C). In addition, there were obvious differences in the number of genes that were mapped to the metabolic pathways. The PI assembly matched a higher number of genes than the PB assembly (PB: 16,687 ± 7090 vs. IL: 484,291 ± 20,103 and IL: 66,130 ± 6714) (Fig. 4D).

### Evaluation of the different assembly techniques predicting natural product biosynthesis genes

The above observations demonstrate the advantage of including long-read data in retrieving long gene sequences from soil DNA samples, which is relatively difficult with the fragmented libraries resulting from the pyrosequencing platforms. The PI sequencing data obtained by correcting the wrong bases in the PB sequence frame and IL sequence is particularly advantageous for predicting the natural product biosynthesis core genes. Modular assembly enzymes, such as polyketide synthases (PKSs), nonribosomal peptide synthetases (NRPSs), and their hybrid enzymes catalyze the most important and diverse classes of natural products that can theoretically code for a nearly infinite diversity of unique structures [8]. The genes that encode PKSs and NRPSs are generally long and consequently pose a major challenge to metagenomic DNA sequencing.

**Fig. 5:**
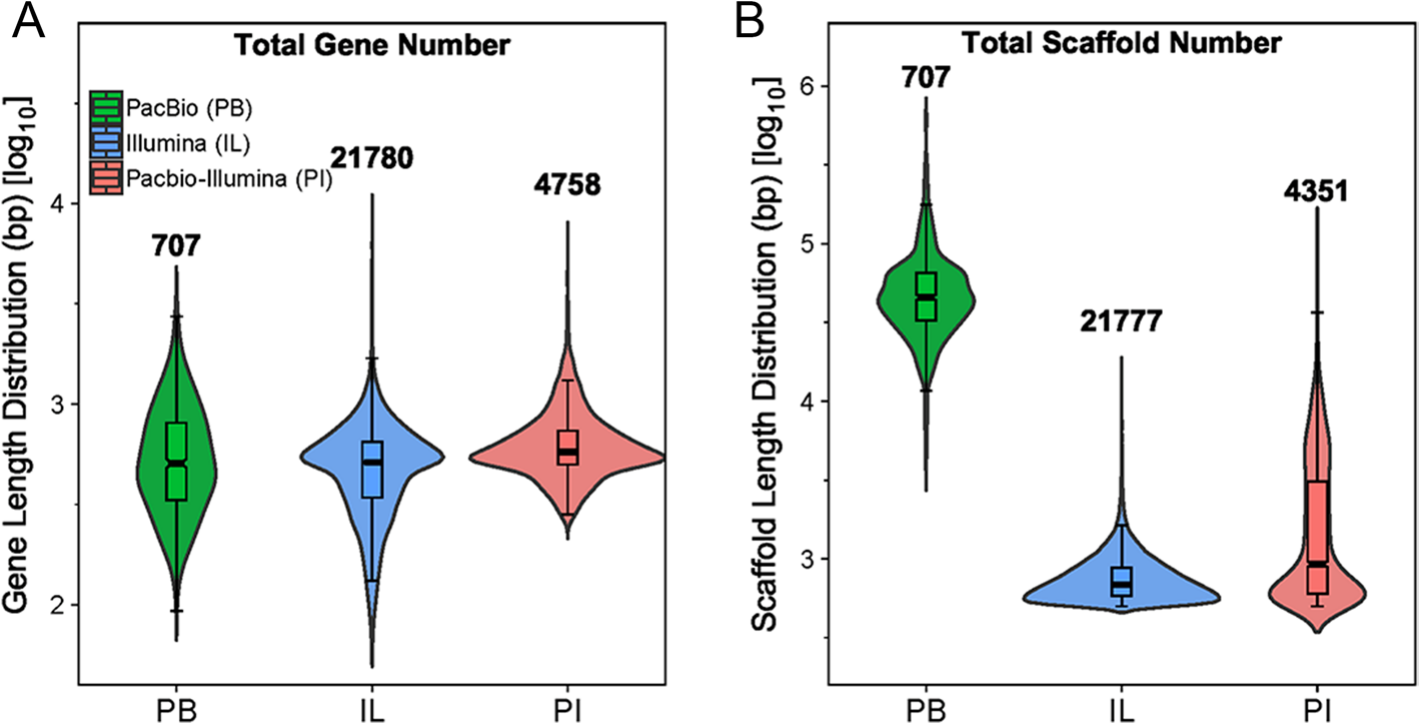
Comparison of the assemblies using only PacBio long reads (PB), only Illumina short reads (IL), and the combination of the two types of data (PI) in the identification of natural product biosynthesis genes including NRPSs, PKSs, and their hybrids. Violin plots show the length distribution of (**A**) natural product biosynthesis genes and (**B**) the gene-containing scaffolds

A total of 707, 21,780, and 4758 predicted genes respectively, were found in the PB, IL, and PI assemblies, using HMM (protein profile Hidden Markov Model) searches of the conserved domains (ketosynthase domain for PKS; adenylation and condensation domains for NRPS) (Fig. 5A). The average value of the relative abundance of PKS and NRPS genes was (2.28 ± 2.09) × 10^− 6^, showing their low abundance and copy numbers. The small number of genes identified using PB data revealed its weakness in recovering low-abundance sequences from the metagenome. However, despite the fact that the total number was low, the quality of gene-carrying scaffolds generated by PB long reads was outstanding compared to IL, as exemplified by the average length (55,139 bp compared to 797 bp), and the number of BGCs (biosynthetic gene clusters) identified by antiSMASH (62 compared to 31). This also showed how difficult it is to obtain the full-length sequences of these long genes by assembling IL short reads into long contigs; instead, combining both PB reads and IL contigs is one solution to balance the sensitivity and gene integrity. As shown in Fig. 5B, the number and length of genes, as well as the gene-containing scaffolds, were significantly improved. The PI assembly generated 4351 gene-carrying scaffolds with an average length of 2.596 kb, within which 122 BGCs were identified by antiSMASH. This was much better than the IL-only contigs (21,777 contigs, average length 797 bp, 31 BGCs) and outcompete PB reads for the number of scaffolds (707 scaffolds).

### Comparison of the microbial diversity

**Fig. 6:**
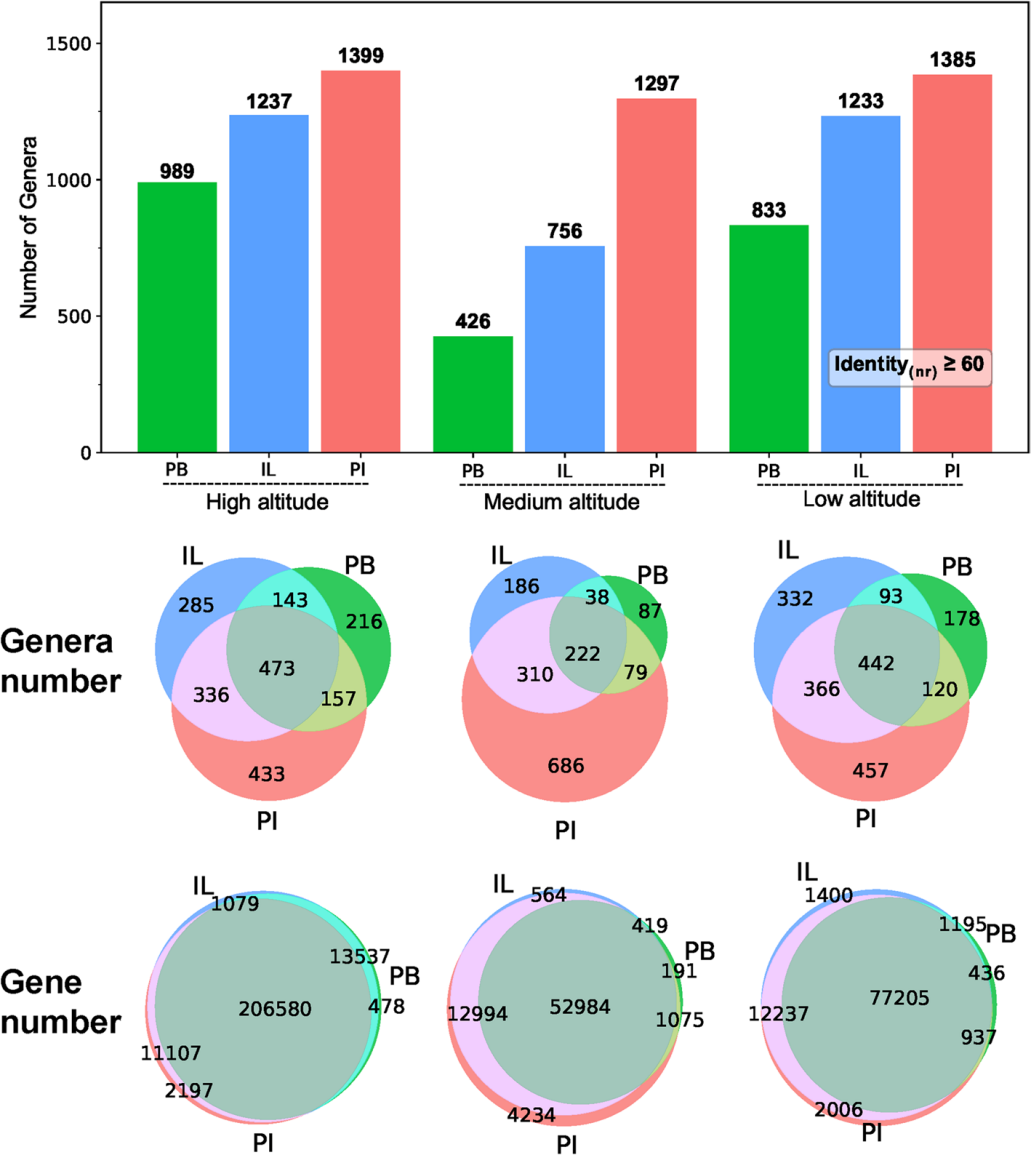
The number of genera predicted by the PB, IL, and PI methods. The predicted gene sequences were annotated by *nr* database, the genera numbers and the corresponding gene sequence numbers were counted after selecting the items whose Identity (nr) was ≥60%

The Tianshan mountain range is a rich source of diverse bacterial and fungal communities. Comparing the three sequencing/assembly methods used in this study in general, the PI assembly was superior relative to the other two methods in both predicting the number of genera and the number of genes (Fig. 6). Based on the number of genera predicted by the three sequencing/assembly methods for the soil samples collected at the different altitudes, in all the assemblies, the high-altitude soil seemed to contain the most genera, followed by the low and medium altitude samples. The number of genera predicted from PI assembly showed a relatively stable trend (Fig. 6) that was higher than those observed in the other two assemblies. According to the Venn diagrams in Fig. 6, the numbers of genera that could only be predicted by PI assembly (433, 686, and 457) were larger than other two assembly methods (IL: 285, 186 and 332 and PB: 216, 87 and 178) indicating that the PI assembly was more sensitive detecting microbial diversity. In addition, in the abundance analysis (PI), we could find that the abundances of the lineages of “Bacteria; Proteobacteria; Alphaproteobacteria; Sphingomonadales (Order); Sphingomonadaceae (Family); Sphingomonas (Genus)” and “Bacteria; FCB group; Gemmatimonadetes; Gemmatimonadetes; Gemmatimonadales (Order); Gemmatimonadaceae (Family); Gemmatimonas (Genus)” are all in the top 7 (Fig. S8), indicated that they are widely distributed on Tianshan mountain area.

In order to know how IL sequencing contributed to PI assembly, we employed the tool MegaBLAST to analyze the differences in gene sequences between IL and PI. The top-score sequences were selected and 95% identity was applied as the threshold to clean the sequences before performing microbial annotation. As shown in Fig. S7, PI assembly could predict many unique genera, and most of the genera annotated by IL assembly were included in the PI genera. The unique genera were matched by few gene sequences, and majority of gene sequences belonged to the genera shared among the assemblies (90.50, 81.97 and 84.64% in high, medium and low altitudes, respectively). These results indicated that PI assembly included the sensitivity of IL assembly and became more sensitive to microbial annotation. Venn figures (Fig. 6, S7) suggested that some microbial information could be lost in both short- and long- metagenomic sequencing and this information will be restored by combining these data.

We also normalized the genera number with clean data size and found that the number of genera predicted by PI per unit clean data (1 Gb) are 106 in high altitude, 116 in medium altitude and 89 in low altitude, which are much smaller than IL (272, 166 and 271 in high, medium and low altitudes, respectively) (Fig. S1B). However, PI ultimately showed better performance and stability in species annotation than IL (Fig. 6). The phenomenon indicated that PI was obviously affected by the size of clean data in the process of standardization, but it is suitable for microbial diversity research.

**Fig. 7:**
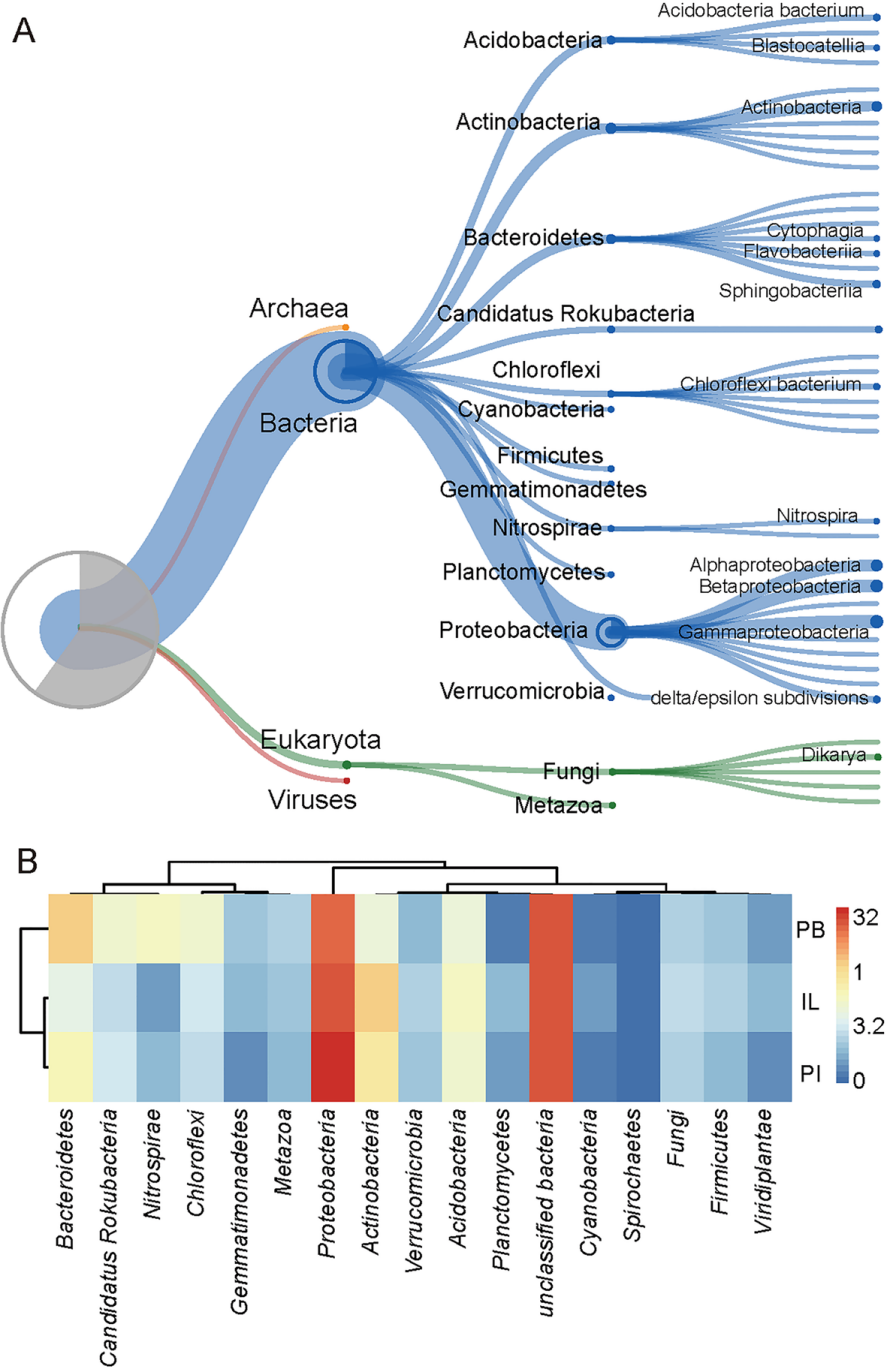
The taxonomic origins of the predicted PKS and NRPS genes in the Tianshan mountain soil samples: (**A**) the major taxonomic classification for genes present at > 0.5%, and (**B**) the distribution of PKS and NRPS genes at the major phylum level (> 0.5%) for the three assemblies

The Unipept pipeline assigned the taxonomic identity on average of 39.8 ± 5.3%, of the NRPS, PKS, and hybrid genes. The majority belonged to *Bacteria* (93.9 ± 2.1%), and the remaining were from *Eukaryota*, *Archaea*, and *Viruses* (Fig.S8). The major taxonomic classifications in the PI with relative abundance of > 0.5% are represented in Fig. 7A and B shows the distribution at the phylum level in the three assemblies. The most abundant genera represented were *Proteobacteria* (32.6 ± 6.8%) and *Actinobacteria* (7.6 ± 3.8%). Indeed, soil-dwelling cultivable *Actinobacteria* and *Proteobacteria*, represented by *Streptomyces* and *Pseudomonas* spp., respectively, have been the most prolific sources of the bioactive natural products [8, 14, 30]. The taxonomic classification also highlighted the prokaryotic phyla *Bacteroidetes* (6.9 ± 4.7%), *Acidobacteria* (4.3 ± 0.8%), *Chloroflexi* (2.9 ± 1.4%), and *Candidatus Rokubacteria* (2.8 ± 1.2%) as well as the eukaryotic phylum *Fungi* (1.3 ± 0.4%) as the potential sources for natural product discovery.

The distribution of natural product (NP) biosynthesis genes at the phylum level varied slightly across the three assemblies (Fig. 7B). In the kingdom *Bacteria*, *Bacteroidetes* was enriched in the PB assembly (12.14%) compared to 3.0% (IL) and 5.7% (PI). In contrast, *Actinobacteria* and *Proteobacteria* were more abundant in the IL and PI assemblies than in the PB assembly.

### Analysis of genes encoding polyketide synthases (PKSs) and non-ribosomal peptide synthases (NRPSs)

In general, PKSs and NRPSs share high levels of sequence identity among the conserved domains such as adenylation (AD) and condensation (C) domains in NRPSs and the ketosynthase (KS) domain in PKSs. In turn, a high degree of identity among these domains predicts that the corresponding biosynthetic gene clusters (BGCs) are involved in the biosynthesis of structurally-related small molecules. By extension, domain sequences with no close relatives might have arisen from BGCs that produce structurally novel classes of metabolites [31]. AD, C, and KS domains have been successfully used as sequence tags to identify and classify NRPS or PKS enzymes in previous studies [5, 13, 16, 18, 19, 32–36], and thus were used in this study to assess the novelty of NP biosynthesis in the sampled soils.

**Fig. 8:**
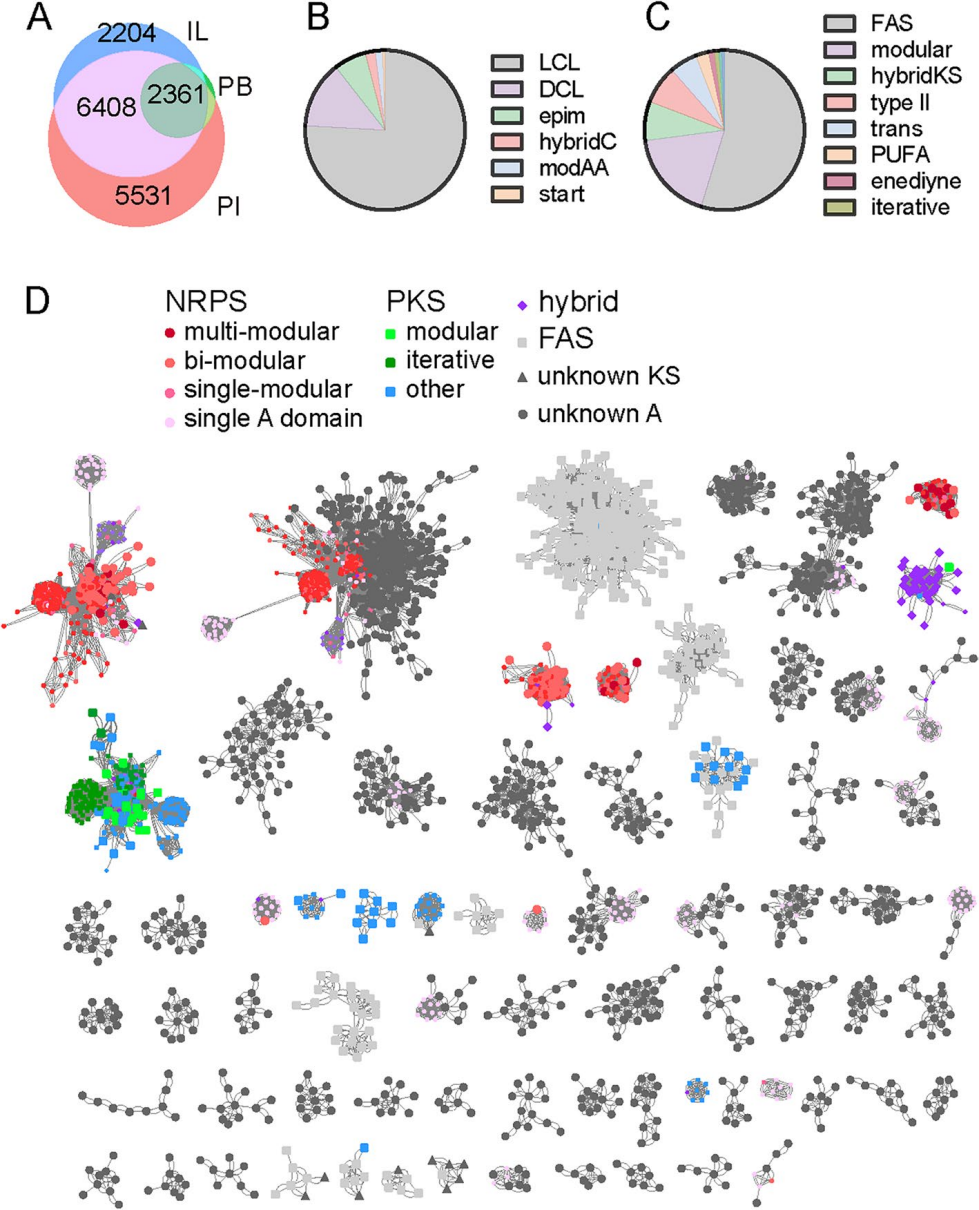
The orthoMCL analysis of the predicted proteins containing A, C and/or KS domains in the three assemblies (**A**), classification of the (**B**) C and (**C**) KS domains based on the NaPDoS phylogenetic analysis, and (**D**) overview of the similarity networks of the AD, C, and KS domains from the Tianshan metagenomes. Network representation of the clades is based on all-versus-all sequence alignments. Each node represents an AD, C, or KS domain, and the edges connect domains with e value < 10^− 60^. The meaning of the node colors and shapes are given in the figure at the top of the panel. Nodes of reference domains from SwissProt have smaller sized labels. Only clades with more than six nodes and at least one node from the metagenomes are shown. LCL, catalyze formation of a peptide bond between two l-amino acids; DCL, link an l-amino acid to a growing peptide ending with a d-amino acid; Epim, epimerization domains change the chirality of the last amino acid in the chain from L- to D-amino acid; modAA, appear to be involved in the modification of the incorporated amino acid; Start, first module of a NRPS. Modular: modular PKS, large multi-domain enzymes consisting of multiple sets of modules, in which each domain is used only once in the synthesis process following the co-linearity rule; hybridKS: biosynthetic assembly lines that include both PKS and NRPS components; PUFA: Polyunsaturated fatty acids (PUFAs), the long chain fatty acids containing more than one double bond, including omega-3-and omega-6- fatty acids; Enediyne: a family of biologically active natural products [37]; iterative: type I iterative PKS which uses the same domain repeatedly to elongate the polyketide chain [38, 39]

The orthoMCL analysis of the predicted proteins containing AD, C and/or KS domains in the three assemblies showed that most (86.6%) were covered by the PI assembly (Fig. 8A). To assign possible catalytic functions to these proteins, the predicted C and KS domain sequences were submitted to the web-based analysis platform NaPDoS (Natural Product Domain Seeker) [37]. Most of the C domains were classified as LCL domains (75.9%) that catalyze the formation of a peptide bond between two l-amino acids, followed by the DCL domains (13.6%) that are located immediately downstream of epimerization domains and thus catalyze the condensation reaction between a d- and an l- amino acid residue (Fig. 8B) [40]. Most KS domains belonged to the fatty acid synthase (FAS) class (54.7%), the modular class (18.2%), and the PKS-NRPS hybrid class (7.9%), while the iterative PKS class represented only 0.9% (Fig. 8C).

A similarity network was constructed using the amino acid sequences from the Tianshan metagenomes and 1651 reference AD, C, and KS domains, and MCL clustering was then used to identify clades of related nodes based on the sequence identity matrix. The resulting profiles of biosynthetic domain sequences from Tianshan mountain soil microbiomes revealed that most of the NRPSs and PKSs from the metagenomes (10,739 out of 12,567 domains) did not cluster with any known NP-encoding genes based on the network and MCL analyses, except for the well-known conserved fatty acid synthase (FAS) and acetyl-CoA ligase clades (Fig. 8D). This suggested the potential to discover novel classes of NP genes from soil DNA libraries. However, most of the predicted sequences were far from satisfactory to enable the recovery of the full biosynthetic cassettes in order to address novel product biosynthesis.

**Fig. 9:**
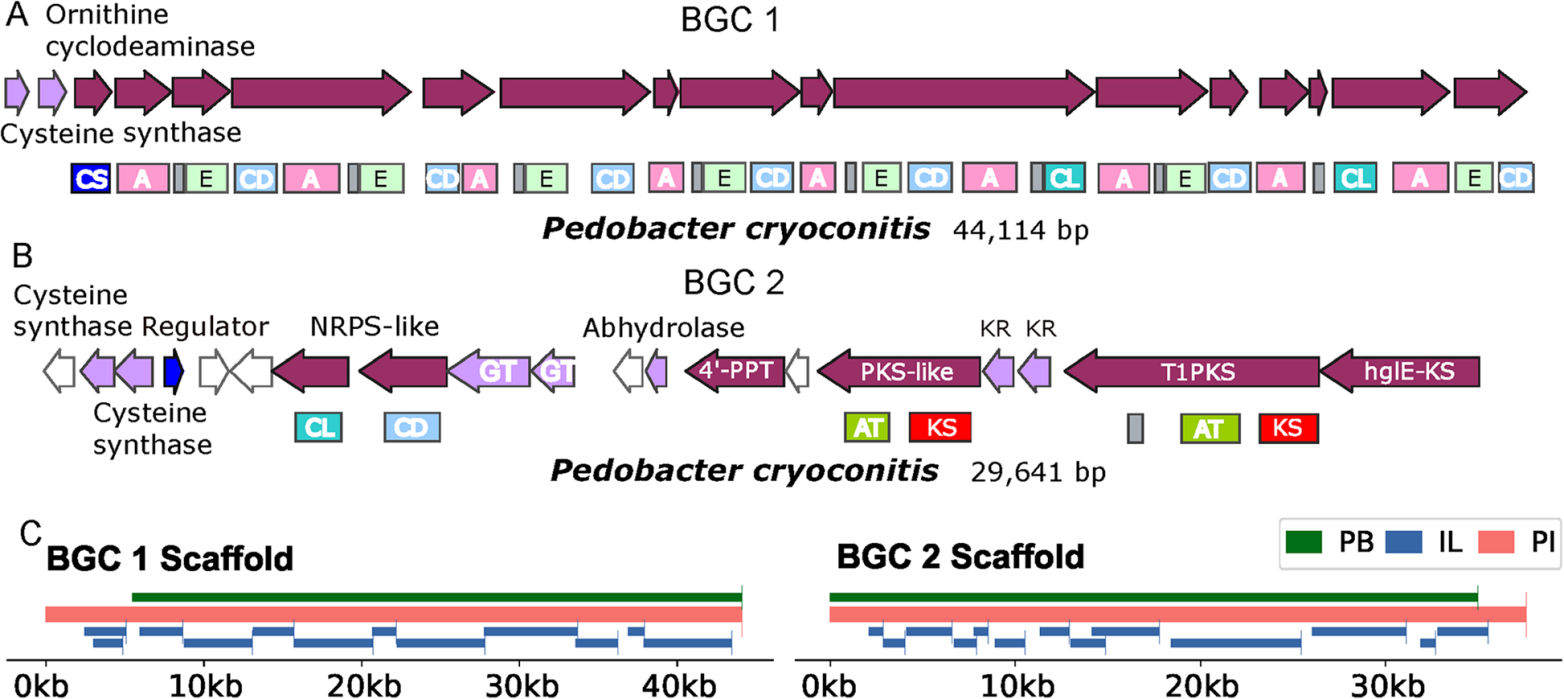
Representative NRPS (**A**) and PKS (**B**) biosynthetic gene clusters predicted from the combined assembly sequences, and (**C**) the map of scaffold coverage for the two gene clusters from each of the three assemblies. BGC, biosynthetic gene cluster; A, adenylation domain; CL, condensation domain linking a L-amino acid to a peptide ending with a L-amino acid; CD, condensation domain linking a L-amino acid to a peptide ending with a D-amino acid; CS, starter condensation domain; E, epimerization domain; GT, glycosyl transferase; 4′-PPT, 4′-phosphopantetheine transferase; hglE-PKS, heterocyst glycolipid synthase-like PKS; KS, ketosynthase; AT, acyltransferase domain; KR, ketoreductase domain

Two examples of the predicted biosynthetic gene clusters are shown in Fig. 9A for NRPSs and Fig. 9B for PKSs. They both originated from *Pedobacter cryoconitis* (Bacteroidetes). Although these clusters span 44.1 Kb and 29.6 Kb, respectively, they both had an incomplete border on one end (upstream in Fig. 9A and downstream in Fig. 9B). The gene cluster in Fig. 9A contains 16 open reading frames (ORFs) that encode the core enzymes (NRPS and NRPS-like) with nine complete modules of adenylation, condensation and peptidyl-carrier protein domains. There are seven modules that also include epimerization domains to convert l-amino acids to their d-isomers, which could then be linked to another l-amino acid residue by the corresponding condensation domains. Single genes for ornithine cyclodeaminase and cysteine synthase are located upstream in this gene cluster and may participate in the modification of the substrate amino acids or peptide products. The adenylation (AD) and condensation (C) domains in this gene cluster are similar to those in proteins that produce linear polypeptide intermediates such as gramicidin [41], surfactin [42], bacitracin [43] based on the similarity network in Fig. 8C. The Fig. 9B cluster (BGC2) is a hybrid of modular PKS and NRPK-like core enzyme and modification enzyme genes. Interestingly, none of the domains could be found in the similarity network in Fig. 8C, suggesting a high probability of a new product. This gene cluster has a heterocyst glycolipid synthase-like PKS (*hglE*-KS) [44, 45] that belongs to a group of assembly-line PKSs frequently found in heterocyst-forming cyanobacteria, and is involved in nitrogen fixation; however, the domain composition of this protein and the protein components of this gene cluster are very different than in the *hgl* cluster. Thus, BGC2 is likely to produce a glycolipid, starting with a reduced polyketide chain with hydroxyl groups catalyzed by a type I PKS, two ketoreductases, and a PKS-like protein. The two types of NRPS-like proteins that contain C_DCL_ and C_LCL_ domains can further link two amino acids to the product which may be processed by the two cysteine synthases. The intermediate product can be processed by the alpha/beta hydrolase targeting one of the carbonyl groups and two glycosyl transferases that likely attach glycosyl groups to the hydroxyl groups on the polyketide chain. A gene of a predicted regulator is located within the cluster, but it is transcribed in the opposite direction to most of the other genes in the cluster. Both BGC 1 and 2 were only covered in the PI (Fig. 9C). This further emphasized the advantage of combining data from both the IL and PB platforms.

## Discussion

Previous [46–48] meta-genome analyses were conducted mostly on sequences generated by IL because it is relatively inexpensive. However, the assembly sequences of this strategy are generally short, which significantly affects the subsequent meta-genomic functional analyses. In this study, the advantages of assembly quality of sequences generated by PB, IL and the two combined was compared using common tools. Our results suggested that the strategy of short read DNA sequencing (IL) could provide more contigs and gene sequences. Based on a large number of short genes, IL assembly provides higher numbers of genes, functional proteins and microbial diversity. The long read DNA sequencing method (PB) in contrast, generates much longer contigs. This is essential to identify genomic sequences of functional genes involved in secondary metabolism, which tend to be long. The combination of the two methods covered up the deficiencies of both IL and PB sequencing, as shown in our and previous studies [11, 12]. In summary, by comparing the three assemblies, we found that the combinatorial assembly is more suitable for metagenomic research.

In addition, we used the gene sequences of three assemblies to annotate the microbial composition, IL assembly produced a large number of gene sequences, but not the most number of genera, while PI assembly uses fewer gene sequences than IL assembly to get the most genera. Comparison of PI and IL assemblies in microbial annotation (Fig. S7) suggested that the PI assembly is more sensitive. Also PI assembly showed more stable performance on microbial prediction than other two assembly methods. PI assembly presented higher numbers of shared microbe genera (PB: 192, IL: 509 and PI: 1113) (Fig. S9). The advantages of PI were not obvious at the order level (Fig. S6). These results suggested that PI assembly was suitable for microbial annotation at the genus level. However, since there were only few soil samples from each altitude locations, more research was needed to obtain reliable conclusions.

In the analysis of predicting natural product biosynthesis genes, PI assembly generally had more and longer genes and scaffolds, which was not as extreme as the other two methods. And it was suitable for the analysis in polyketide synthases (PKSs), nonribosomal peptide synthetases (NRPSs) (Fig. 9). PI assembly could generate more and longer scaffold sequence than other assemblies.

For a complex soil sample, based on our research results, PI assembly was a better choice, which could 1) get longer scaffolds/genes than IL; 2) obtain more scaffolds/genes number than PB; 3) produce more stable numbers of microbial annotation, and 4) is more suitable for the analysis of natural product biosynthesis genes.

We also found that the assembly quality highly depends on the bioinformatics tools. In this research, IDBA and metaSPAdes assembly methods were compared with the same data. Both assemblies had similar N50 values and contigs length distributions (Fig. S10C-D). However, the total contig number and total contig length of metaSPAdes assemblies were higher than the corresponding IDBA assemblies (Fig. S10A-B). After using MegaBLAST, we found the mapped sequences by best bit score and did further research. Regardless of the altitude of the samples, the metaSPAdes dataset provides most of the gene set of IDBA dataset and a large part of additional genes (Fig. S10E-G). Therefore, we concluded that the metaSPAdes dataset provides a metagenomic assembly and this dataset was used to represent IL and used for following analysis. The comparison of IDBA and metaSPAdes on the IL sequencing data indicated that further development in bioinformatics tools used for long-read assembly probably can also improve the results and worth investigation in subsequently study, such as the Canu [49–51], Flye [52–54], MetaFlye [52], Marvel [55], and MaSuRCA [56]. In addition, the strategy of polishing the assembly sequences, such as NextPolish [57], POLCA [58], Pilon [59], and the special tool for gene prediction, are also capable to improve sequence quality, gene prediction and functional meta-genome of the PB and PI, which probably further support our suggestion that combining long and short-read DNA sequencing data is an effective strategy to explore the soil metagenomics.

## Conclusions

In this study, we used three soil samples collected from three different altitudes to evaluate the performance of three high-throughput DNA sequencing/assembly strategies. The third-generation platform (PB) gave long contigs and relatively intact genes, although it provide a smaller proportion of the total gene set present in the soil metagenome. The second-generation sequencing platform (IL) gave the highest sensitivity with respect to the genes, but shorter assembled contigs and predicted genes. The assembly that combined the PB and IL reads had the advantages of both the individual assemblies, i.e., sequencing sensitivity and gene integrity, respectively. Natural product biosynthesis genes were used as an example to evaluate the three different assembly techniques. The result showed that the PI method also has an advantage over the other two methods in that long PKS and NRPS genes could be detected in the soil metagenomes. Additionally, we found many novel classes of NP genes in the Tianshan soil environmental DNA libraries that can be studied in detail in the future.

## Methods

### Soil sample collection and processing

Soil samples were collected from a low altitude area (latitude and longitude: 43.2052 N, 87.1153E, altitude: 2200 m), a medium altitude area (latitude and longitude: 43.11835 N, 87.00049E, altitude: 2750 m) and a high-altitude area (latitude and longitude: 43.11826 N, 86.83936E, altitude: 3700 m) in Ürümqi Glacier No. 1 in the Tianshan Mountains (Xinjiang, China) (Fig. 1A). We delineated a 4 m × 4 m area at the sampling points and performed five-point sampling. Samples were collected with sterile equipment from the top 10 cm of the soil layer, then mixed and placed in the same sample bags, stored with ice in bags for transport to the laboratory, and immediately frozen at − 80 °C upon arrival.

### Library construction and DNA sequencing

For PacBio DNA sequencing, libraries with insert sizes of 20 kb were constructed using the SMRTbell Template Prep Kit (Pacific Biosciences, Menlo Park, CA, USA). For short-read sequencing, libraries were constructed with the Illumina TruSeq Nano DNA Library Prep Kit (Illumina, San Diego, CA, USA). Library construction with the detailed protocols as shown in the published references [60]. The Sequel instrument was programmed to load and sequence the sample on PacBio SMRT cells v3.0 (PacBio p/n 100–171-800), acquiring one movie of 360 min per SMRT cell on the PacBio Sequel platform. MagBead loading (PacBio p/n 100–125-900) method was used to improve the enrichment of the larger fragments. For the samples sequenced with Illumina technology, the short-insert 400 bp library was sequenced on an Illumina Hiseq 2000 instrument at Beijing Genomics Institute (Shenzhen, Guangdong, China).

### Filtering of the sequencing data

For the PacBio data, subreads were filtered using the following parameters: filtered subreads with adapters; removed the polymerase reads with quality < 0.8; filtered out subreads < 1000 bases in length. For the Illumina data, the clean reads were filtered using the following parameters: filtered reads with adapters; trimmed reads with two low-quality bases at the 5′ end and three low-quality bases at the 3′ end; removed reads with > 10% N (unknown) bases; filtered duplicated reads due to polymerase chain reaction amplification; discarded reads with > 50% low-quality bases (Q20 < 20).

### Sequence assembly

The metagenome assembly of all soil samples was carried out as follows: 1) PB assembly; MetaFlye was used for the de novo assembly of subreads with the designated parameters (flye --pacbio-raw subreads.fa --genome-size 377 m --meta). 2) IL assembly; SPAdes (version 3.12.0) [61] was used for the de novo assembly of short paired-end reads with the designated parameters (−m 20,000 -t 8 -k 21, 33, 55, 77, 99, 127 --phred-offset 33 --meta). 3) PI assembly; SPAdes (version 3.13.0) was used for the de novo assembly of metagenomes by combining the PacBio subreads and the Illumina short reads using the designated parameters (−-meta -m 1000 -t 60 -k 21, 33, 55, 77, 99, 127), and subsequently the assembly sequences were corrected with the SOAPsnp (parameters: -u -t -z @ -Q i -q) and SOAPindel programs (parameters: -c 3 -h 1 -u 2 -m 2) [62] using the short reads.

### Gene prediction, annotation and microbial annotation

Protein-coding genes in the assembled metagenomes were predicted de novo using MetaGeneMark [63] with the default parameters. The general annotation of the predicted proteins was performed with the following programs: putative functional annotations were retrieved from the NCBI *nr* database using BLASTP to identify the best homologues, COG (Clusters of Orthologous Groups of proteins), eggNOG (evolutionary genealogy of genes: Non-supervised Orthologous Groups) [64] and the InterProScan 5 (incorporated InterPro, Gene Ontology, and KEGG pathway annotation) [65] database were used to determine the functional categories of the predicted proteins. For microbial annotations, putative microbial information were retrieved from the NCBI nr database by using BLASTN. The top-score sequences were selected and 60% identity were applied as the threshold and used TAXONKIT to extract microbial lineage for subsequent analysis.

### Prediction and taxonomic analysis of polyketide synthases (PKSs) and nonribosomal peptide synthetases (NRPSs)

Adenylation (AD), condensation (C), and ketosynthase (KS) domain-containing proteins were identified using the AMP-binding (PF00501), condensation (PF00668), and ketosynthase (PF00109) domain models from PFAM (http://pfam.sanger.ac.uk/) and the search tool hmmersearch in the HMMER package (http://hmmer.org/). Taxonomic annotation of the predicted protein sequences of polyketide synthases (PKSs) and nonribosomal peptide synthetases (NRPSs) were performed using Unipept software [66]. Kaiju web software was also used for the taxonomic annotation of PKS- and NRPS-encoding genes [67].

### Classification and annotation of PKSs and NRPSs

The KS and C domains from the PKSs and NRPSs were submitted to NaPDoS (The Natural Product Domain Seeker, a bioinformatic tool for the rapid detection and analysis of secondary metabolite genes) [37] for classification [37]. Similarity networks were constructed by all-versus-all sequence alignment of the predicted protein sequences of the conserved domains, i.e., KS for PKSs, AD and C for NRPSs. A total of 1651 AD, C, and KS domains were extracted from proteins in the SwissProt database with known products and used as references. Markov clustering (MCL) was used to identify clades of related nodes based on the sequence identity matrix. Based on empirical data from previous metagenomic analyses [32, 68, 69], the homology cutoff was set to an expected value (E-value) of < 10^− 60^. PKSs and NRPSs from the network analysis were associated with potential metabolite families.

## Supplementary Information

**Additional file 1.**

## Data Availability

All sequencing data has been deposited at the NCBI under BioProject PRJNA658179.

## Abbreviations

PB: Third-generation long-read sequencing platforms (PacBio)
IL: Second-generation / short-read sequencing platforms (Illumina)
PI: Combined sequencing assembly (mixture of PB and IL)
DNA: DeoxyriboNucleic Acid
NPs: Natural products
BGCs: Biosynthetic gene clusters
Kb: Kilobases
COG: Clusters of Orthologous Groups of proteins
eggNOG: Evolutionary genealogy of genes: Non-supervised Orthologous Groups
KEGG: Kyoto Encyclopedia of Genes and Genomes
PKs: Polyketide synthases
NRPSs: Nonribosomal peptide synthetases
AD: Adenylation
C: Condensation
KS: Ketosynthase
NaPDoS: Natural Product Domain Seeker
HMM: Hidden Markov Model
FAS: Fatty acid synthase
ORFs: Open reading frames
*hglE*-KS: heterocyst glycolipid synthase-like PKS
