# Supplementary material for: Combined assembly of long and short sequencing reads improve the efficiency of exploring the soil metagenome
Source: BMC Genomics. 2022 Jan 7;23:37. doi: 10.1186/s12864-021-08260-3 (PMC8742384; doi:10.1186/s12864-021-08260-3)
Supplement: Supplementary file 1 — Additional file 1. [file 12864_2021_8260_MOESM1_ESM.pdf]

Supplementary materials

Figure S1 | the contig number (**A**) and genera number (**B**) were normalized by the clean data size (unit: Gb).

Note: the clean data size of PI (Combined assembly) is the sum of the clean data size of the other two methods(PB, PacBio; IL, illumina), the detailed clean data size was showed in Table **S**1.

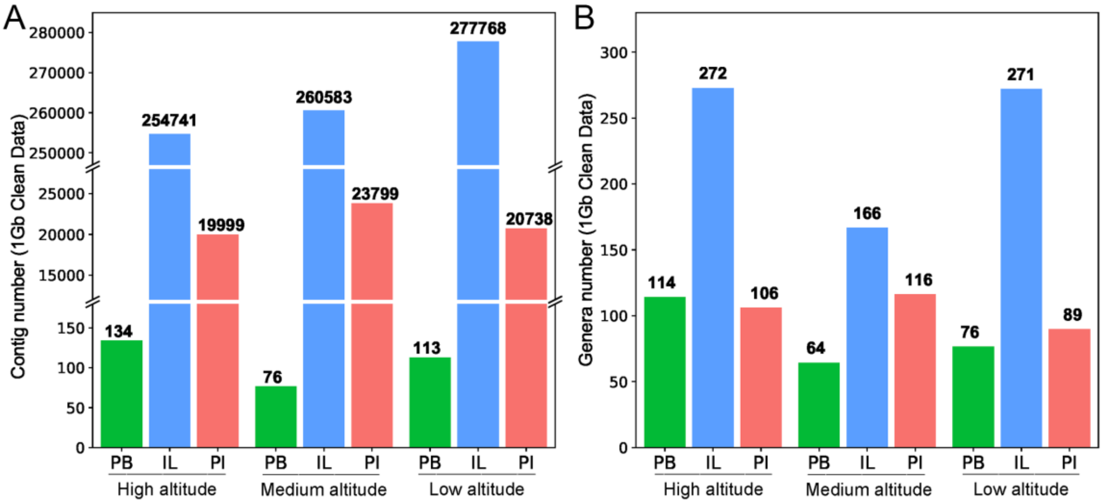

Figure S2 | the distribution of gene length in samples at different altitudes。

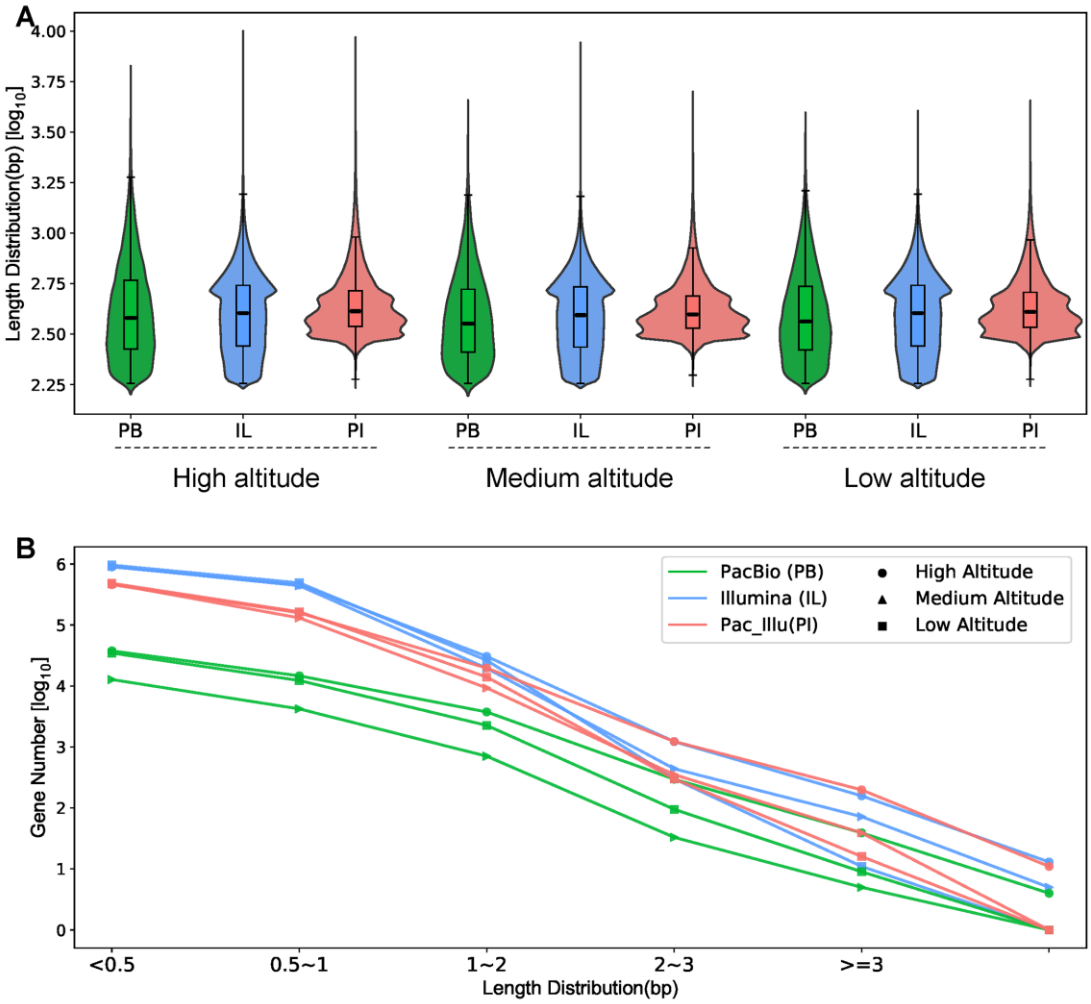

Figure S3 | the predicted protein sequences were used to perform orthologous gene analysis, and Venn diagrams are drawn to show the overlap between the three assembly techniques. Here are the results of ortholog genes analysis of different samples.

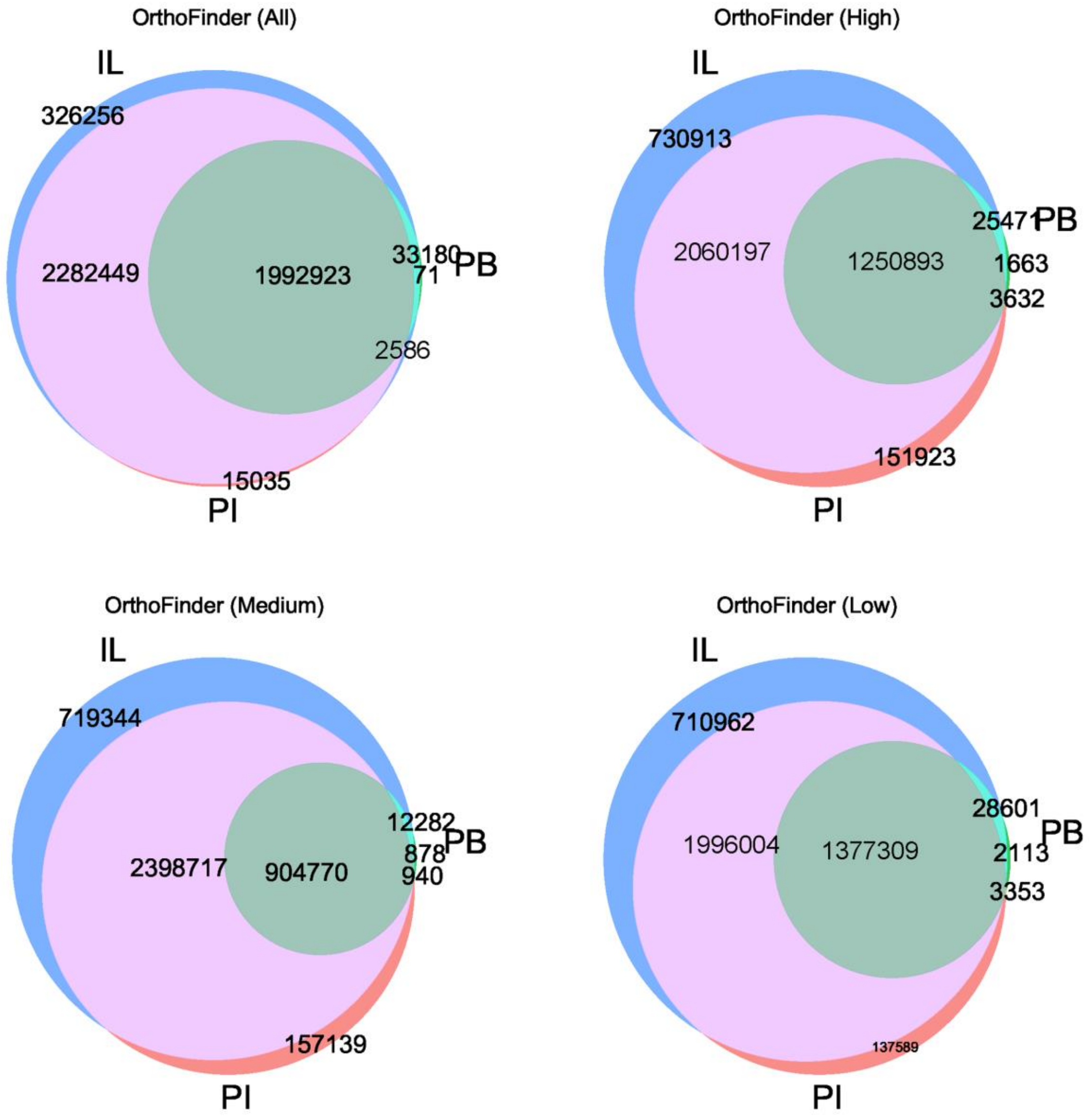

Figure S4 | the Corresponding Gene Number of Each COG's Proteins

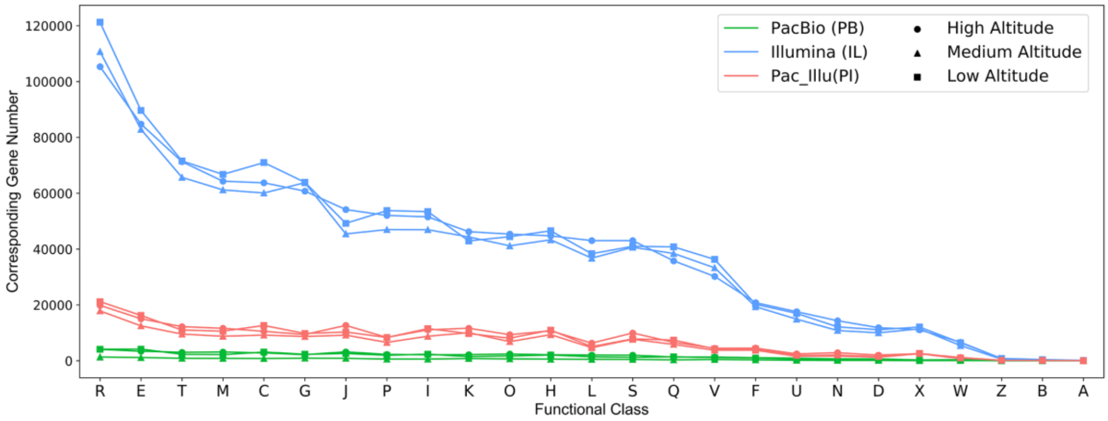

Figure S5 | Gene Functional Annotation of Each Method (KEGG)

(A) Metabolic pathways predicted by PacBio

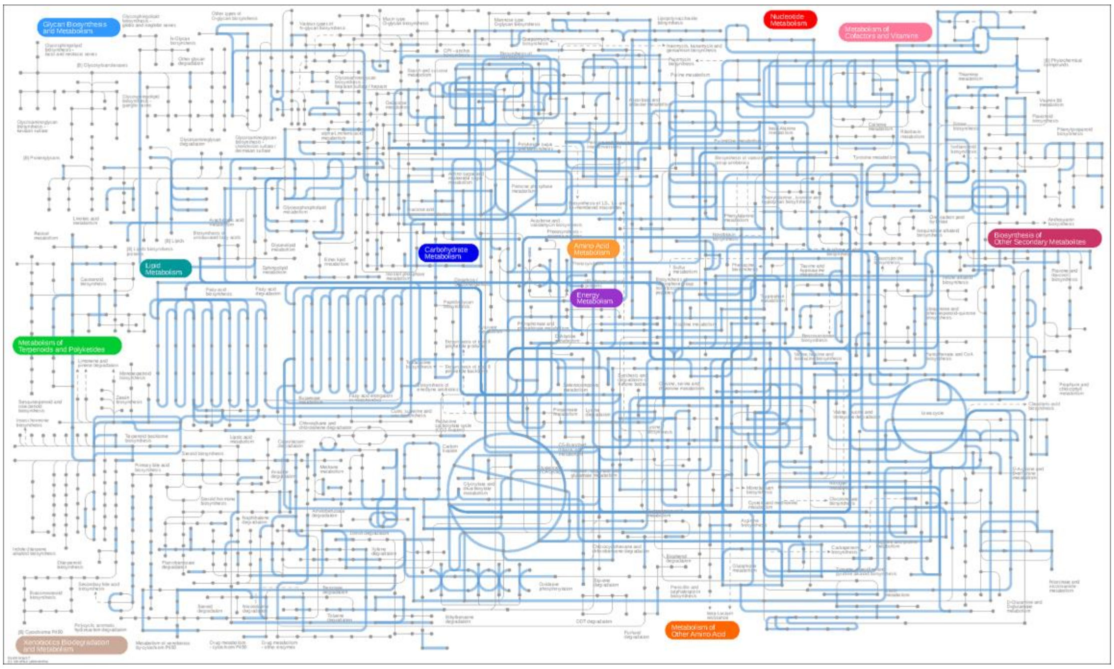

(B) Metabolic pathways predicted by Illumina

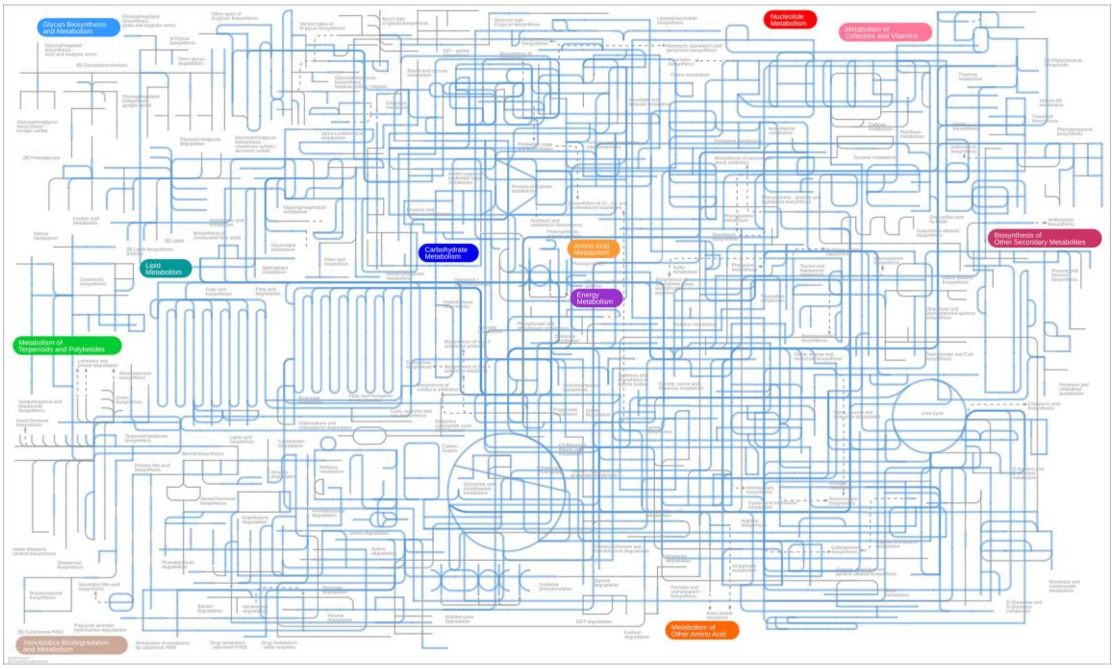

(C) Metabolic pathways predicted by PacBio_Illumina

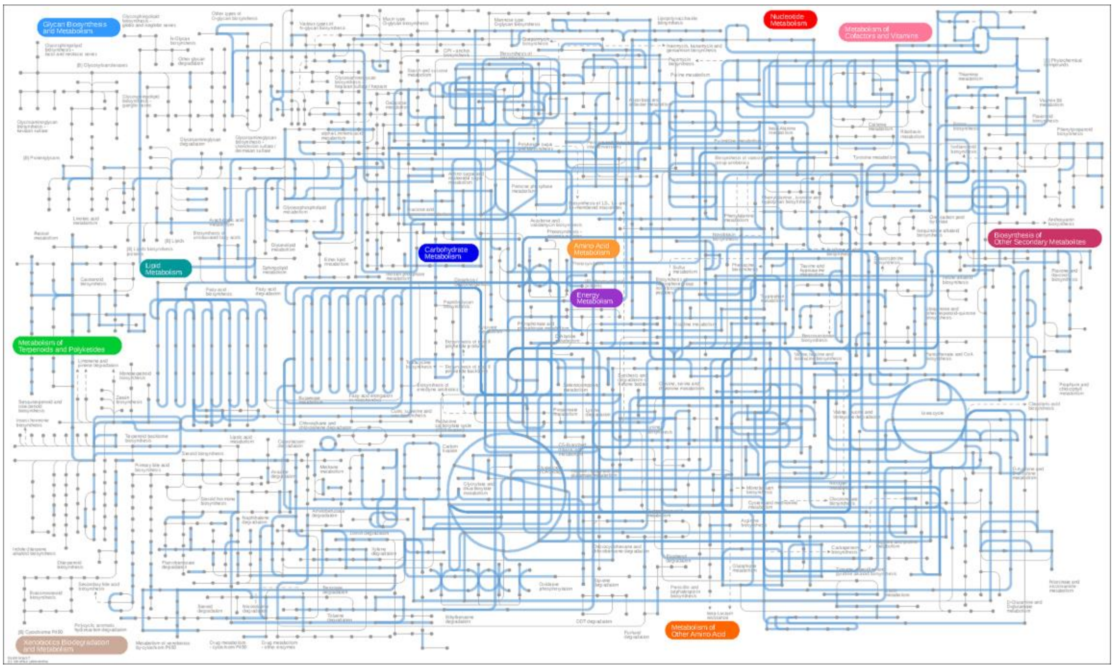

Figure S6 | the taxonomic diversity and abundance of three levels (genera, families and orders)

The number of genera (or families or orders) shown in the column chart, and the stacked histogram shown the most abundant 20 genera (or families or orders) and the "LOW ABUNDANCE" which composed of other genera (or families or orders).

Genera:

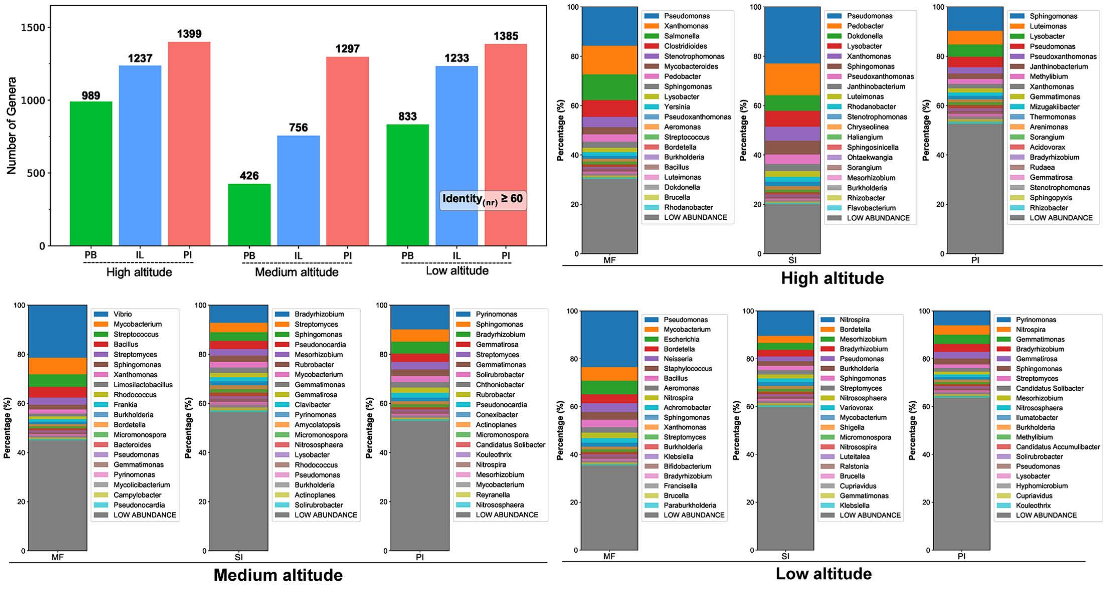

Family:

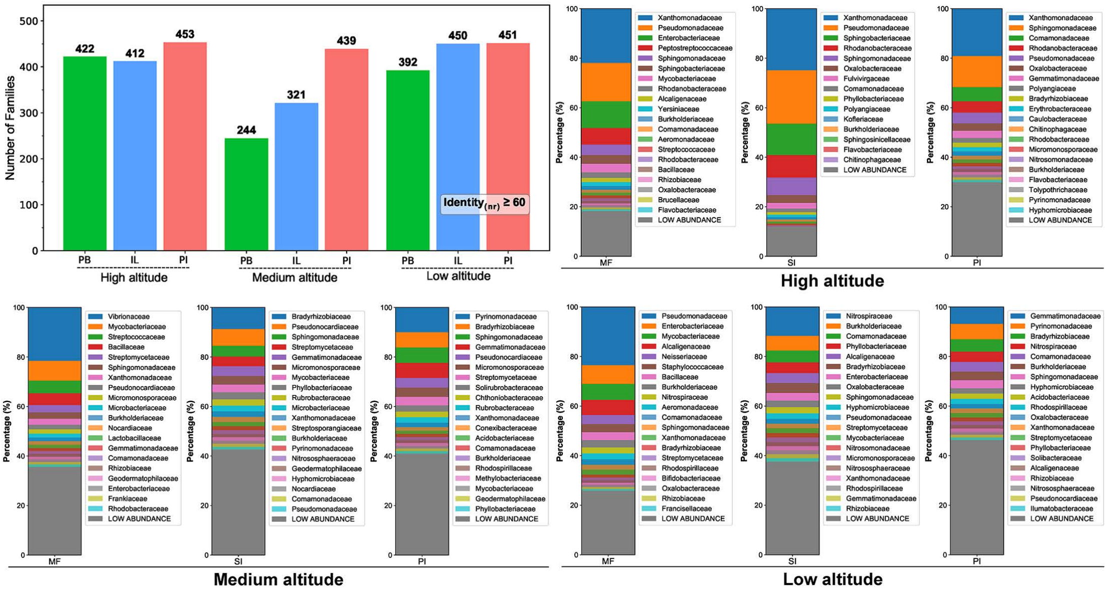

Order:

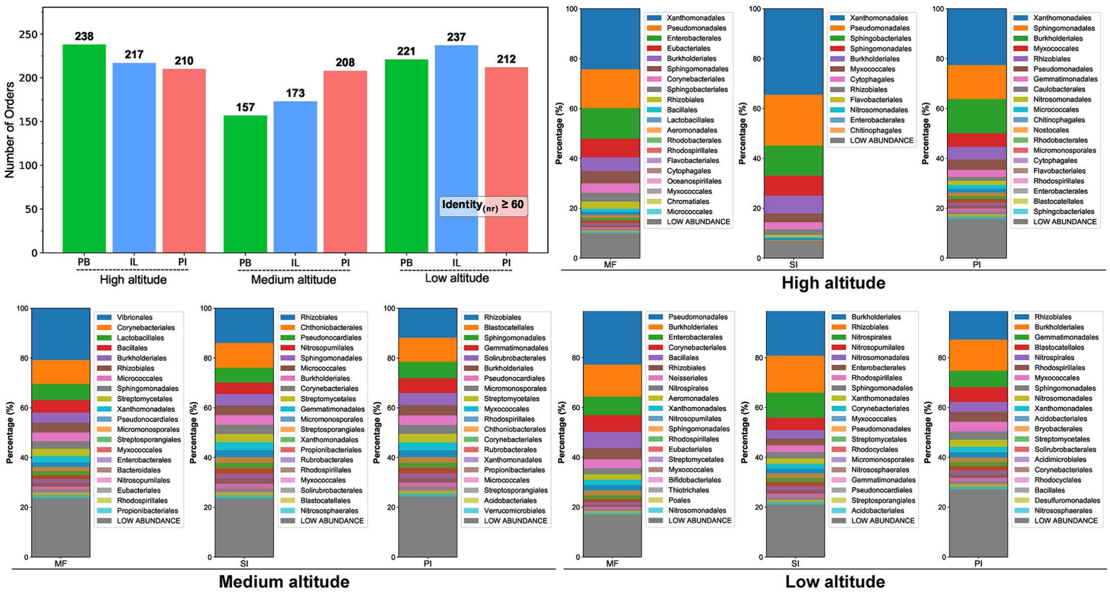
Number of Orders
238
217
210
157
173
208
221
237
212
PB
IL
PI
High altitude
Medium altitude
Low altitude
Identity(nr) ≥ 60
Percentage (%)
MF
SI
PI
High altitude
Medium altitude
Low altitude

Figure S7 | Comparison of PI and IL assembly in microbial annotation

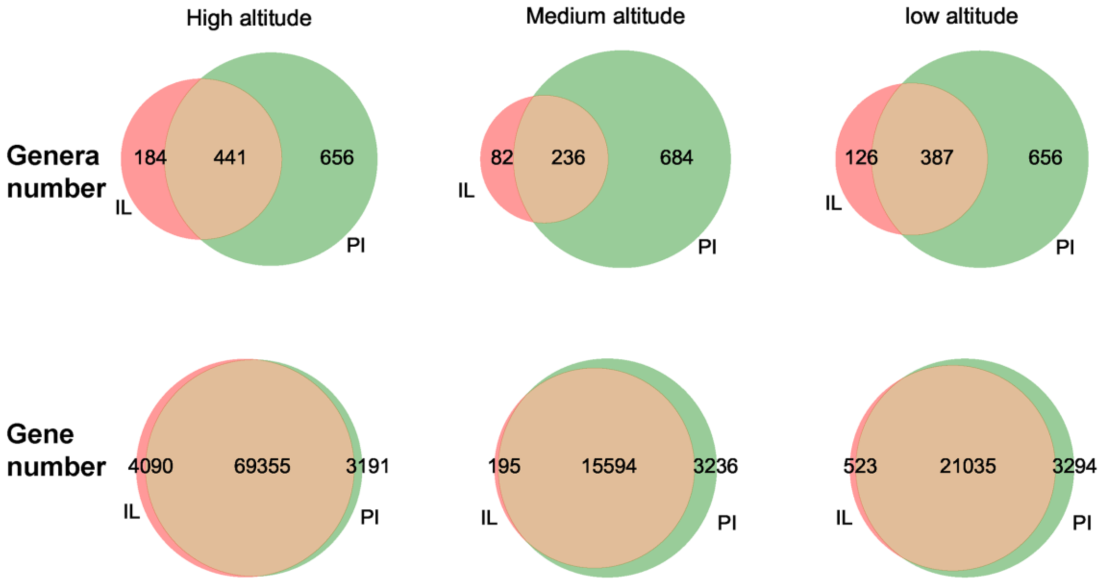

Figure S8 | the abundance at different taxonomic levels in the analysis of secondary metabolism

Note: " LOW ABUNDANCE " consisted of species with abundance less than 0.5%.

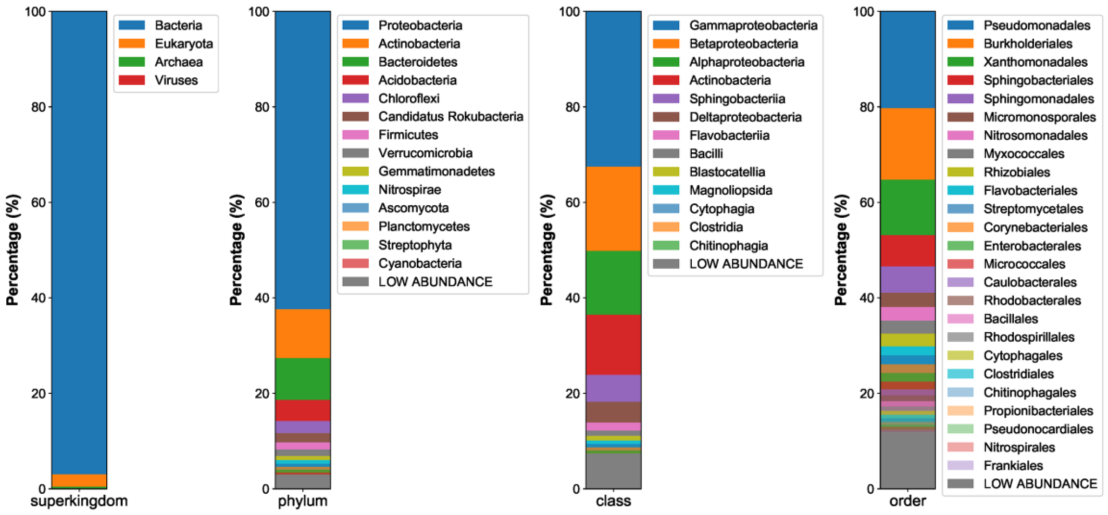

Figure S9 | the relationship of genera among three altitude locations

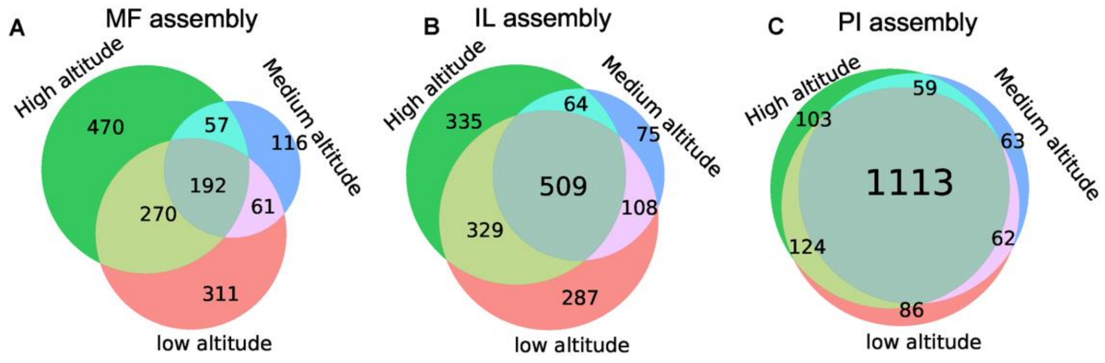

Figure S10 | Comparison of IDBA and metaSPAdes in IL assembly

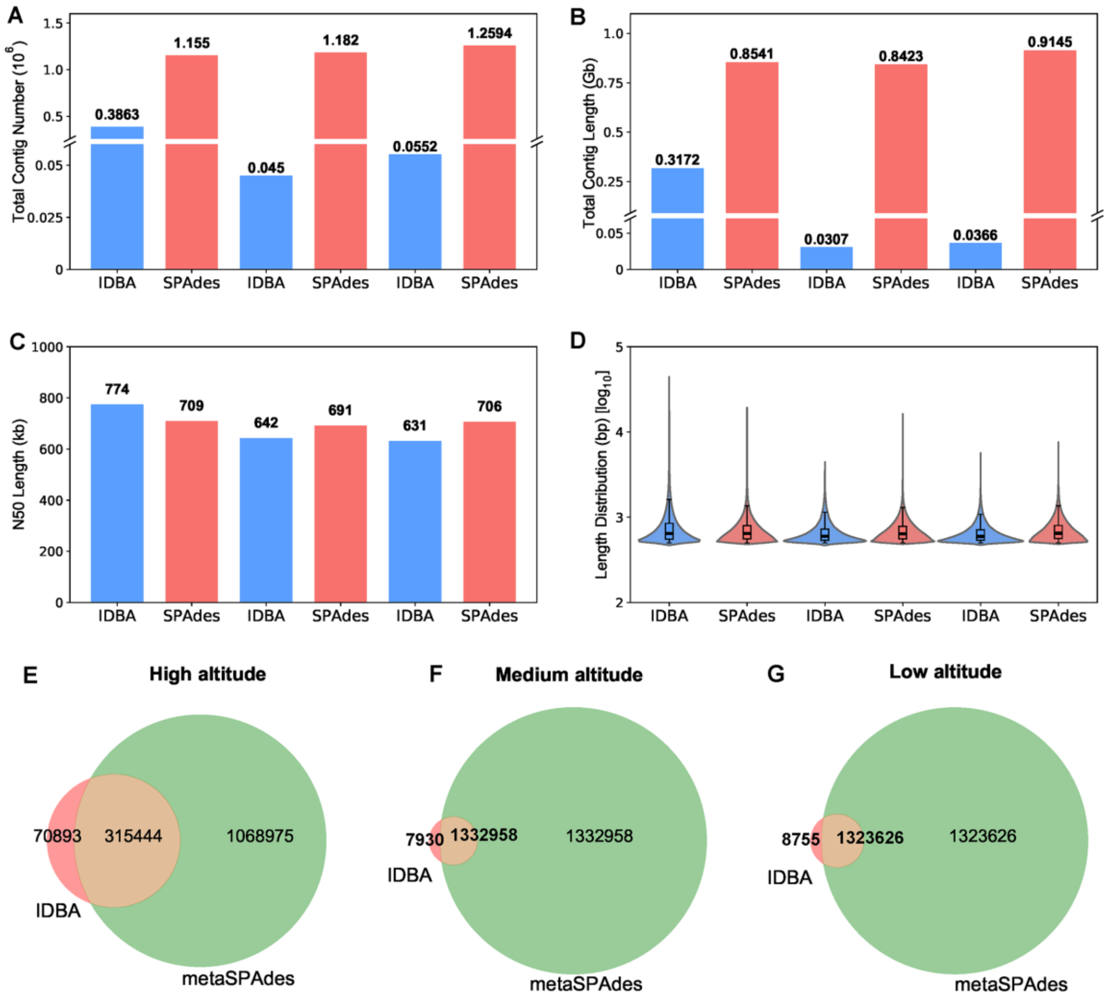

Table S1 | General information on the metagenome sequences

Parameters	Altitude	Methods		
		PacBio	Illumina	PacBio_Illumina
Clean Data Length (Mb)	High	8,662	4,534	Mix
	Medium	6,616	4,536	Mix
	Low	10,897	4,534	Mix
Clean Data Reads	High	1,191,948	32,756,894	Mix
	Medium	871,813	32,617,286	Mix
	Low	1,583,970	32,523,820	Mix
GC Content (%)	High	61.32	64.20	62.01
	Medium	65.16	65.52	64.27
	Low	63.50	64.88	63.69
N90 (bp)	High	24,243	531	574
	Medium	22,619	529	546
	Low	20,370	532	572
Min (bp)	High	530	500	500
	Medium	1,823	500	500
	Low	139	500	500
Max (bp)	High	607,831	18,606	125,626
	Medium	201,162	15,115	59,996
	Low	215,241	7,338	30,022
≤1kb (%)	High	0.77	84.73	67. 59
	Medium	0.00	90.74	71.66
	Low	0.32	89.14	63.47
Average (≤1kb) (%)		0.45	89.53	67.31

Table S2 | Total Gene Length and Distribution

	Altitude	Methods		
		PacBio	Illumina	PacBio_Illumina
Total Gene Length (bp)	High	27,415,281	616,818,174	303,042,687
	Medium	7,773,057	591,292,800	271,568,379
	Low	22,225,644	649,938,207	304,547,418
Sum		57,413,982	1,858,049,181	879,158,484

Table S3 | Gene Length Distribution

	Length	Methods		
		PacBio	Illumina	PacBio_Illumina
Gene Length Distribution	<0.5kb	84,994	2766188	1,413,875
	0.5~1kb	31,158	1386383	453,113
	1~2kb	6,705	76329	42,938
	2~3kb	421	1960	1,882
	≥3kb	53	254	260

Table S4 The detailed function classes in Fig. 3**A** are described as follows:

Classes of COG	Detailed function classes of COG
A	RNA processing and modification
B	Chromatin structure and dynamics
C	Energy production and conversion
D	Cell cycle control, cell division, chromosome partitioning
E	Amino acid transport and metabolism
F	Nucleotide transport and metabolism
G	Carbohydrate transport and metabolism
H	Coenzyme transport and metabolism
I	Lipid transport and metabolism
J	Translation, ribosomal structure and biogenesis
K	Transcription
L	Replication, recombination and repair
M	Cell wall/membrane/envelope biogenesis
N	Cell motility
O	Posttranslational modification, protein turnover, chaperones
P	Inorganic ion transport and metabolism
Q	Secondary metabolites biosynthesis, transport and catabolism
R	General function prediction only
S	Function unknown
T	Signal transduction mechanisms
U	Intracellular trafficking, secretion, and vesicular transport
V	Defense mechanisms
W	Extracellular structures
X	Mobilome: prophages, transposons
Z	Cytoskeleton

The x ticks in panel A and panel C are arranged in descending order of PI data.
